# Narrow Complex Tachycardia With Alternating R-R intervals. What is the mechanism?

**Published:** 2006-10-01

**Authors:** David Foo, KS Ng, Lucy Qu, Antonio Sutandar

**Affiliations:** 1 The Heart Institute, Tan Tock Seng Hospital, Singapore; 2 The Heart Institute, National University Hospital, Singapore

## Case Presentation

A 25 year old man underwent electrophysiology study for recurrent symptomatic paroxysmal palpitations. There were no documented episodes of supraventricular tachycardia on surface ECG.  Delta waves were absent on baseline ECG.

**Figure 1:**
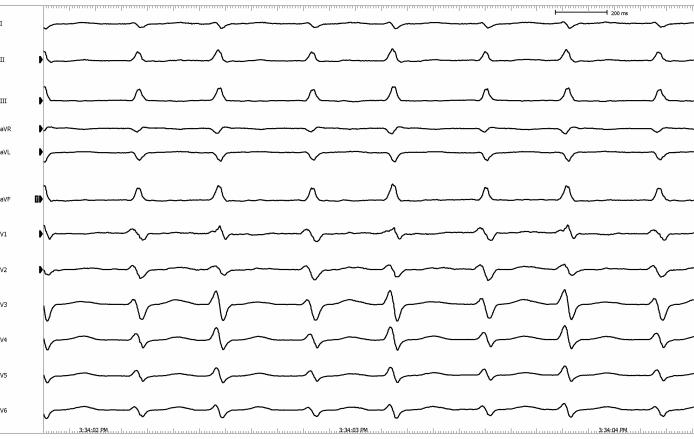
Surface ECG showing supraventricular tachycardia with alternating R-R intervals

**Figure 2:**
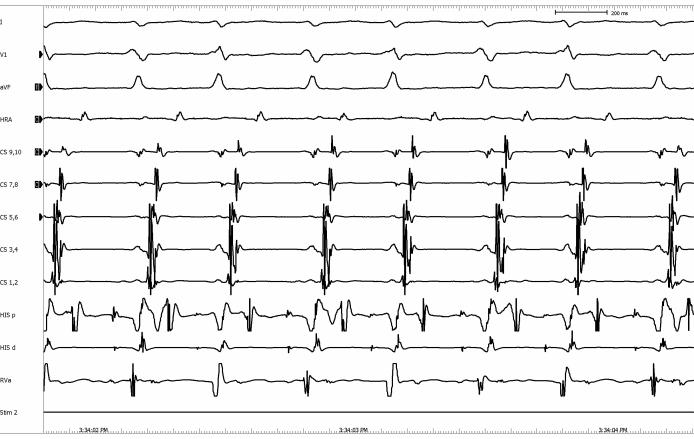
Intracardiac electrogram. HRA = high right atrial electrogram; HIS p and HIS d = proximal and distal HIS-bundle electrogram respectively; CS 1-2, CS 3-4, CS 5-6, CS 7-8, CS 9-10 = distal bipole, bipole 3-4, bipole 5-6, bipole 7-8, proximal bipole respectively; RVa = right ventricular apex electrogram; Stim 2 = stimulus channel

A decapolar catheter was placed in the coronary sinus with the distal and proximal pair of electrodes configured as CS 1-2 and CS 9-10 respectively. Quadripolar catheters were positioned in the high right atrium, His-bundle region and right ventricular apex. During straight atrial pacing with a drive-cycle length of 340ms, the following arrhythmia was induced (Figure 1). The corresponding electrogram is shown in Figure 2. What is the mechanism?

## Commentary

The surface ECG revealed a narrow complex supraventricular tachycardia with alternating R-R intervals at 310ms and 360ms (Figure 1). The intracardiac electrogram showed a macro-reentrant circuit with the earliest retrograde atrial activation located at CS 1-2. Therefore, the patient exhibited a concealed left sided accessory pathway as the retrograde limb of the tachycardia circuit.

 On close inspection of the intracardiac electrogram, two different alternating A-H intervals were observed, which presented as alternating R-R intervals on the surface ECG.

The A-H intervals were 110ms and 160ms consecutively. We deduced that the antegrade limb of this macro-reentrant circuit involved both the fast and slow AV nodal pathways alternating with each other.

Radiofrequency ablation of the left lateral pathway via a retrograde aortic approach was performed. The tachycardia was successfully terminated. Further electrophysiology studies confirmed the presence of dual AV nodal pathways. There were no further inducible arrhythmias even with isoproterenol infusion. The slow pathway was thus not ablated.

The mechanism for this supraventricular tachycardia with alternating R-R intervals involved two alternating antegrade limbs (fast and slow AV nodal pathways) with different conduction times and a common retrograde concealed left sided accessory pathway. This electrophysiological mechanism had been postulated previously based on appearance of the surface ECG without intracardiac recordings [1].
